# A Reliability Fault Diagnosis Method for Diesel Engines Based on the Belief Rule Base with Data-Driven Initialization

**DOI:** 10.3390/s25165091

**Published:** 2025-08-16

**Authors:** Huimin Guan, Guanyu Hu, Hongyao Du, Yuetong Yin, Wei He

**Affiliations:** 1 School of Computer Science and Information Engineering, Harbin Normal University, Harbin 150025, China; 2024300705@stu.hrbnu.edu.cn (H.G.); 18845127666@163.com (H.D.); 2 Key Laboratory of Equipment Data Security and Guarantee Technology, Ministry of Education, Guilin University of Electronic Technology, Guilin 541004, China; 3 College of Information and Electrical Engineering, China Agricultural University, Beijing 100083, China; yuetong@cau.edu.cn

**Keywords:** diesel engines, fault diagnosis, belief rule base (BRB), data-driven

## Abstract

Diesel engines serve as critical power sources across transportation and industrial fields, and their fault diagnosis is essential for ensuring operational safety and system reliability. However, acquiring sufficient and effective operational data remains a significant challenge due to the high complexity of the systems. As a modeling method that incorporates expert knowledge, the belief rule base (BRB) demonstrates strong potential in resolving such challenges. Nevertheless, the reliance on expert knowledge constrains its practical application, particularly in complex engineering scenarios. To overcome this limitation, this study proposes a reliability fault diagnosis method for diesel engines based on the belief rule base with data-driven initialization (DI-BRB-R), which aims to improve modeling capability under conditions of limited expert knowledge. Specifically, the approach first employs fuzzy c-means clustering with the Davies–Bouldin index (DBI-FCM) to initialize attribute reference values. Then, a Gaussian membership function with Laplace smoothing (LS-GMF) is developed to initialize the rule belief degrees. Furthermore, to guarantee the reliability of the model optimization process, a group of reliability guidelines is introduced. Finally, the effectiveness of the proposed method is validated through an example of fault diagnosis of the WD615 diesel engine.

## 1. Introduction

As critical power sources, diesel engines are extensively utilized across various domains, including transportation, industry, and power generation. The operational safety of diesel engines is directly linked to human safety and the stable performance of equipment [1]. Nevertheless, due to factors such as high temperature, high pressure, vibration, and fluctuations in combustion efficiency during prolonged operation, diesel engines are susceptible to sudden failures if potential faults are not identified promptly [2,3]. Such failures can pose dual threats, potentially resulting in both personal injury and significant economic losses. Therefore, achieving reliable and accurate fault diagnosis for diesel engines is not only essential for maintaining operational stability and extending service life but also vital for enabling condition monitoring, preventive maintenance, and intelligent control [4,5]. The development of a scientifically sound fault diagnosis framework can significantly reduce failure rates, lower maintenance costs, and enhance overall operational efficiency and management capability, thereby providing strong technical support for safe and reliability engineering operations [6,7,8].

Current research on diesel engine fault diagnosis can be broadly categorized into three methodological paradigms: data-driven models, knowledge-driven models, and hybrid approaches. Data-driven models rely primarily on historical operational data to automatically extract features and construct predictive and diagnostic frameworks through statistical analysis and machine learning techniques [9]. For example, Gao et al. proposed a fault diagnosis method for marine diesel engine piston rings based on a long short-term memory (LSTM) neural network optimized via an improved beluga whale optimization algorithm [10]. The experimental results demonstrated enhanced fault identification accuracy. Li et al. introduced a multimodal input transformer-based deep convolutional neural network to detect ignition misfire faults in diesel engines [11]. By integrating multidimensional features, the method effectively suppressed environmental noise and exhibited superior noise robustness compared with conventional approaches. Wang et al. combined infrared thermography with a convolutional neural network (CNN) to develop an image-based fault diagnosis technique, leveraging temperature distribution patterns [12]. Through adaptive histogram equalization and SoftMax-based classification, their method outperformed other deep learning models in fault identification tasks. Yang et al. proposed an end-to-end diesel engine fault diagnosis framework based on a multi-attention convolutional neural network, which incorporates improved attention modules and self-attention mechanisms to enhance feature extraction capabilities [13]. Experimental results revealed an accuracy exceeding 97% across multiple fault categories, with high diagnostic efficiency. Chen et al. proposed an incremental fault diagnosis approach with bias correction (IFD-BiC), addressing the challenge of sequential data acquisition in diesel engine diagnostics [14]. By introducing distillation loss and bias correction mechanisms, the method maintains prior knowledge while learning new fault tasks. Experiments confirmed its strong diagnostic accuracy and incremental learning performance under varying operating conditions. Despite their performance advantages, data-driven models heavily depend on large volumes of labeled data, exhibit limited interpretability, and often suffer from poor generalizability.

Knowledge-driven models focus on leveraging physical principles, expert experience, logical reasoning, and engineering laws to diagnose engine faults. These models do not rely on large-scale data and are fundamentally rooted in the philosophy of “understanding systems through human knowledge.” Yang et al. developed a three-dimensional coupled vibration model to diagnose crankshaft crack faults in diesel engines, proposing a primary–secondary criterion coordination strategy that integrates experimental and simulation data for crack feature identification [15]. The results demonstrated that the method effectively detected crankshaft cracks, enhancing diagnostic accuracy and practical applicability. Xu et al. proposed an improved thermoeconomic diagnostic approach by combining traditional diagnostic techniques with component performance characteristic curves, allowing for the separation of intrinsic and induced fault impacts [16]. A validation of a multi-fault diesel engine experiment revealed accurate identification of all the faulty components and assessment of their economic consequences. Coelho et al. applied fault tree analysis (FTA) to diesel engine diagnostics, constructing logical fault structures and generating rule-based diagnostics, which were further combined with neural networks for state inference [17]. Their approach achieved clear discrimination of operating conditions and demonstrated strong consistency and practical viability. Knežević et al. utilized FTA to model and quantitatively assess faults in the turbocharging system of marine diesel engines [18]. By simulating various fault scenarios, their method contributed to more informed maintenance planning, improved system reliability, and enhanced turbocharger efficiency. Du et al. established a four-cylinder diesel engine dynamic model incorporating a cracked crankshaft via the finite element method and analyzed vibration characteristics and crack effects under different working conditions [19]. The results showed that waterfall plots could effectively distinguish various fault types, providing theoretical support for vibration-based diagnostics. Nevertheless, knowledge-driven methods heavily depend on expert input, which limits their adaptability to complex nonlinear systems. Additionally, the modeling and computational requirements are often substantial, restricting their scalability and real-time applicability.

Hybrid-driven models combine the advantages of knowledge-driven and data-driven approaches by fusing expert knowledge with data features, overcoming the limitations of single methods and enhancing the comprehensiveness and reliability of diagnosis. Zhan et al. proposed a diesel engine fault diagnosis method based on optimized variational mode decomposition (VMD) and an improved convolutional neural network (CNN) [20]. The experimental results demonstrated superior classification accuracy compared with conventional methods, highlighting the approach’s potential for preventive maintenance applications. Guo et al. developed a transfer learning approach based on a multi-scale, multi-view domain adversarial network to address the lack of fault labels under varying diesel engine operating conditions [21]. When applied to diesel engine diagnostics, the method achieved an accuracy of 96.58%, outperforming other models and improving fault detection capabilities in intelligent marine engines. To tackle the challenge of similar fault manifestations across different diesel engine components, Jiang et al. utilized a k-nearest neighbor–mutual information method to filter out low-correlation thermodynamic parameters [22]. These were then fused using t-SNE-based dimensionality reduction, significantly improving diagnostic accuracy. Li et al. proposed a health assessment approach for marine diesel engine turbochargers based on a zero-dimensional engine model coupled with machine learning [23]. The method employs an attention-enhanced convolutional long short-term memory network to predict key parameters, constructed a health index using the Mahalanobis distance, and performs probabilistic interval prediction for quantitative health evaluation. Li et al. introduced a progressive adaptive sparse attention-guided incremental knowledge feature mining method [24]. By fusing fault-sensitive knowledge features with latent representations, this approach achieves remarkable diagnostic performance under complex operating conditions and limited data scenarios.

Diesel engines are complex mechanical systems that typically operate under highly dynamic and harsh conditions [25]. Their structural complexity, nonlinear behavior, and strong coupling between components pose significant challenges to fault diagnosis. In addition, when using sensors to collect data during engine operation, it is often affected by noise, missing values, and external interference, resulting in highly ambiguous and uncertain information related to faults [26]. This uncertainty and nonlinearity reduce the accuracy and effectiveness of traditional diagnostic methods, making it difficult to accurately identify fault patterns. Therefore, diesel engine fault diagnosis requires advanced modeling techniques that can effectively handle incomplete, inaccurate, and uncertain information to ensure accurate and reliable identification of faults under complex operating conditions [27].

As a typical hybrid-driven model, the belief rule base (BRB) is an intelligent expert system that integrates the characteristics of both knowledge-driven modeling and data-driven models [28]. The BRB framework combines fuzzy theory, Dempster–Shafer (D-S) evidence theory, and the “IF-THEN” rule, introducing belief degree parameters into the IF-THEN rule [28,29]. This enables it to effectively address challenges caused by uncertainty, incomplete information, and fuzziness in complex systems [30]. By effectively integrating qualitative knowledge and quantitative information, the BRB is capable of handling uncertain information and demonstrates outstanding modeling capabilities [31]. Based on these advantages, the BRB is particularly suitable for complex fault diagnosis tasks involving small samples, strong uncertainty, and high noise levels. Therefore, the BRB shows significant applicability and advantages in the field of diesel engine fault diagnosis. In the fault diagnosis of diesel engines, the BRB can perform fault diagnosis by integrating expert knowledge and observation data. Firstly, an initial BRB model is constructed based on expert knowledge, including antecedent attributes, reference values, belief degrees, and other parameters. When inputting observation data of diesel engines, the model calculates the matching degree between the data and the corresponding reference values through fuzzy membership functions. Then these matching degrees are combined with the rule weights assigned by experts to generate rule activations. Finally, the rules are fused using the Evidential Reasoning (ER) algorithm to obtain the belief degrees of the fault state provided by the expert. This belief degrees are then weighted with the corresponding fault state to obtain the final utility value, which is the state of the diesel engine. Recently, BRBs have been widely applied for diesel engine fault diagnosis. For instance, Yin et al. proposed an interpretable belief rule base safety state assessment model with reverse causal reasoning, which effectively enhances both the diagnostic accuracy of diesel engines and the interpretability and traceability of the model’s results [32]. Liu et al. proposed a reliability belief rule base model, which effectively improved the accuracy and reliability of diesel engine health status assessment by setting reliability guidelines and introducing a constrained whale optimization algorithm for parameter optimization [33]. Chang et al. proposed a belief rule base concurrent fault prediction method based on custom attribute weights and trade-off analysis [34]. Taking a marine diesel engine as an example, the method is verified to have high accuracy in identifying both single and concurrent faults, and its performance is superior to that of traditional BRB methods and other comparative models. Xu et al. proposed an expert system model consisting of multiple parallel belief rule base subsystems [35]. The experimental results demonstrates that this approach achieves superior accuracy and stability compared to other advanced models in diesel engine fault diagnosis. Li et al. proposed a new method for fault diagnosis based on attribute reliability using a belief rule base that considers multiple fault features [36]. By introducing attribute reliability into the matching degree calculation, this method effectively minimizes the interference of unreliable information and enhances the accuracy of diesel engine fault diagnosis.

However, the existing BRB models generally rely on the initial reference values and belief degrees supplied by experts. For diesel engine systems characterized by structural complexity, environmental variability, and noisy operating conditions, it is particularly challenging to acquire comprehensive and accurate expert knowledge. Under conditions of insufficient or uncertain expert input, constructing a reliable initial BRB model becomes difficult, thereby increasing the complexity and management costs of fault diagnosis and limiting the method’s applicability in real-world scenarios [37]. To address this issue, some researchers proposed automated BRB models to obtain the key parameters required for model construction, thereby alleviating the dependence on expert knowledge. For example, Zhang et al. proposed a method for behavior prediction of complex systems, termed the structural adaptive BRB (SA-BRB), which employs an improved error-constrained K-means++ algorithm to mine historical data for reference value sets [38]. Wu et al. developed an automated belief rule base model for pathologic complete response prediction in gastric cancer [39]. This approach uses information gain ratio with partial expert knowledge to determine reference values and applies a tabular strategy to initialize rule belief degrees. Although these methods alleviate the problem of insufficient expert knowledge to some extent, they still rely on partial expert knowledge. When expert knowledge is extremely scarce, model performance may still be affected. Moreover, the precision and stability of fault diagnosis for diesel engines directly affect equipment safety and operational efficiency. Reliability research can improve the performance of diagnostic models under complex working conditions and interference conditions, enhance their noise resistance and adaptability, and ensure accurate and reliable fault identification. This is crucial for achieving timely maintenance and extending engine service life. Therefore, to cope with these issues, this study proposes a reliability fault diagnosis method for diesel engines based on a belief rule base with data-driven initialization (DI-BRB-R). This method aims to construct a more scientific, reasonable, and highly reliable fault diagnosis model to improve the accuracy and reliability of diesel engine system fault identification. With the support of a data-driven initialization mechanism, the model exhibits stronger adaptability when facing complex working conditions and uncertain information. This adaptability effectively reduces the risk of misjudgment and missed diagnosis in the fault diagnosis process, thereby reducing maintenance costs and improving system operation efficiency and management level. At the same time, it also provides strong technical support for the safe and stable operation of diesel engine systems. In this method, attribute reference values and belief degrees are determined through data mining techniques, enhancing the model’s initialization quality and reducing reliance on expert knowledge. In addition, a group of reliability guidelines are introduced to guarantee the consistency and reliability of the optimization process. Therefore, the proposed DI-BRB-R model can effectively address the limitations of expert dependent modeling and improve diagnostic reliability, making it very suitable for the challenging conditions of diesel engine fault diagnosis. The primary contributions of this work include the following:

(1) To address the challenge of insufficient expert knowledge in diesel engine fault diagnosis, a DI-BRB-R model is developed.
(2) To ensure the effectiveness of the model, a fuzzy c-means clustering with the Davies–Bouldin index (DBI-FCM) and a Gaussian membership function with Laplace smoothing (LS-GMF) are proposed, which are used to initialize attribute reference values and belief degrees, respectively.
(3) To ensure the reliability and consistency of the optimization process, a set of reliability guidelines is introduced to guide the optimization of parameters, thereby maintaining a balance between accuracy and reliability of the model.

## 2. BRB Basics and Problem Formulation

### 2.1. BRB Basics

On the basis of the RIMER methodology [29], the *k*th rule in the BRB can be formulated as follows:

(1) $$\begin{array}{ll} R_{k}: & IF(X_{1}\;is\;A_{1}^{k})\wedge (X_{2}\;is\;A_{2}^{k})\wedge \ldots \wedge (X_{T}\;is\;A_{T}^{k})\\ & then\;y\;is\;\{(D_{1},\beta_{1,k}),\ldots ,(D_{N},\beta_{N,k})\}\\ & with\;rule\;weight\;\theta_{k}(k=1,2,\ldots ,L)\\ & and\;attribute\;weights\;\delta_{1},\delta_{2},\ldots ,\delta_{T} \end{array}$$

where $X_{i}(i=1,\ldots ,T)$ represents the set of T antecedent attributes. $A_{i}^{k}$ represents the reference value of the *i*th antecedent attribute in the *k*th rule. $D_{i}(i=1,\ldots ,N)$ represents the set of N possible outcomes. $\beta_{i,k}(i=1,\ldots ,N)$ denotes the belief degrees corresponding to the N outcomes in the *k*th rule. $\theta_{k}(k=1,\ldots ,L)$ denotes the rule weight of the *k*th rule. $\delta_{i}(i=1,\ldots ,T)$ represents the weight of the *k*th antecedent attribute.

The ER algorithm is typically employed as the inference engine of the BRB [40]. The reasoning mechanism is explained in the following steps:

(1) Input transformation: The input information is converted into the following belief distribution:

(2) $$S(x_{i})=\{(A_{i,j}^{k},a_{i,j}^{k}),i=1,\ldots ,T;J=1,\ldots ,J_{i}\}$$

where $A_{i,j}^{k}$ represents the *j*th reference value of the *i*th attribute in the *k*th rule. $a_{i,j}^{k}$ denotes the relevant belief degree.

(2) The calculation of matching degree: The matching degree is computed as follows:

(3) $$\alpha_{k}=\prod_{i=1}^{T}{\left(a_{i}^{k}\right)}^{{\bar{\delta }}_{i}},\;{\bar{\delta }}_{i}=\frac{\delta_{i}}{\underset{i=1,2,\ldots ,T}{\max}\{\delta_{i}\}}$$

where $\alpha_{k}$ denotes the matching degree of the *k*th rule. ${\bar{\delta }}_{i}$ denotes the normalized attribute weight.

(3) Activation weight calculation: The activation weight is computed as follows:

(4) $$\omega_{k}=\frac{\theta_{k}\alpha_{l}}{\sum_{l=1}^{L}\theta_{l}\alpha_{l}},\theta_{k}\in [0,1]$$

where $\omega_{k}$ indicates the activation weight of the *k*th rule.

(4) Rule aggregation: The final belief degrees are obtained through rule aggregation based on the ER algorithm:

(5) $$\beta_{n}=\frac{\mu [\prod_{k=1}^{L}(\omega_{k}\beta_{n,k}+1-\omega_{k}\sum_{j=1}^{N}\beta_{j,k})-\prod_{k=1}^{L}\left(1-\omega_{k}\sum_{j=1}^{N}\beta_{j,k}\right)]}{1-\mu \left[\prod_{k=1}^{L}\left(1-\omega_{k}\right)\right]}$$

(6) $$\mu ={\left[\sum_{n=1}^{N}\prod_{k=1}^{L}(\omega_{k}\beta_{n,k}+1-\omega_{k}\sum_{j=1}^{N}\beta_{j,k})-(N-1)\prod_{k=1}^{L}\left(1-\omega_{k}\sum_{j=1}^{N}\beta_{j,k}\right)\right]}^{-1}$$

where $\beta_{n}(n=1,2,\ldots ,N)$ indicates the belief degree associated with the *n*th outcome.

Thus, the final belief distribution of the BRB is given by

(7) $$S(\overset{⌢}{x})=\{(D_{n},\beta_{n})|n=1,2,\ldots ,N\}$$

where $\overset{⌢}{x}$ indicates a set of input data.

(5) Utility computation: The expected utility is computed using the following formula:

(8) $$u(S(\overset{⌢}{x}))=\sum_{n=1}^{N}u(D_{n})\beta_{n}$$

where $u(D_{n})$ indicates the utility of $D_{n}$.

### 2.2. Problem Formulation

When applying the BRB model for diesel engine fault diagnosis, three key challenges must be addressed to improve the model’s construction capability and diagnostic effectiveness:

Problem 1: How to construct a BRB model in the absence of sufficient expert knowledge

(1) Reference value initialization: The antecedent attributes being assigned reasonable reference values is crucial for guaranteeing the BRB model’s effectiveness. Properly defined reference values not only enable accurate system characterization but also enhance model interpretability [37]. Traditional approaches typically rely on expert knowledge to set these reference values. However, in scenarios where expert input or prior information is limited, selecting appropriate reference values becomes subjective and even problematic. Therefore, one of the core focuses of this paper is to propose a method for initializing antecedent reference values when expert input is lacking. The initialization process can be formulated as

(9) A=F(x)

where x indicates the set of evaluation indicators in the fault diagnosis model; F(⋅) denotes the function used to initialize reference values.

(2) Belief degree initialization: The belief degree assigned to each rule in the BRB model reflects the confidence associated with its output. Accurately assigning belief degrees is essential for capturing the uncertainty inherent in complex systems. However, when expert knowledge is limited, determining appropriate belief degrees becomes a major challenge, potentially compromising the model’s fault identification capability. Therefore, exploring effective ways to initialize belief degrees under expert-scarce conditions is another key issue in constructing a reliable BRB model. The initialization process is defined as

(10) β=P(A,x)

where β indicates the initial belief degree; P(⋅) indicates the belief initialization function.

Problem 2: How to ensure reliability during the model optimization process

Optimizing BRB parameters, such as attribute weights, rule weights, and belief degrees, can significantly enhance diagnostic accuracy. However, the optimization process is often stochastic in nature, which may lead to unreliable outputs and reduce the model’s applicability in real-world engineering scenarios [41]. To address this issue, a group of reliability guidelines is introduced to regulate the optimization procedure and ensure that the resulting model achieves a balance between accuracy and reliability. These guidelines can be expressed as

(11) $$guidline:\{g|g_{1},g_{2},\ldots ,g_{t}\}$$

(12) $$\kappa_{best}=optimize(\beta ,\delta ,\theta ,g)$$

where g denotes reliability guidelines. t denotes the number of reliability guidelines.

Problem 3: How to construct the DI-BRB-R model for diesel engine fault diagnosis

To address the above two problems, this study proposes DI-BRB-R. The proposed method aims to achieve high-reliability diagnostics under conditions of limited expert knowledge. The overall model structure is defined as

(13) $$y=f(x,A,\kappa_{best},g)$$

where y represents the diagnostic output. f(⋅) denotes the DI-BRB-R model.

## 3. Construction of DI-BRB-R

### 3.1. Reference Values Initialization

When building a BRB fault diagnosis model, initializing reference values for antecedent attributes plays a fundamental role. These reference values define the fuzzy partitions of input variables and serve as the basis for input transformation and rule activation. Traditionally, reference values are determined based on expert knowledge. However, in practical applications, such expertise may be limited, particularly when dealing with large-scale historical datasets or operating in data-rich but knowledge-scarce environments. Consequently, determining reference values in a scientific and objective manner remains a key challenge in BRB model development [39].

To cope with this issue, this paper proposes a reference value initialization method based on DBI-FCM, aiming to enhance the expressiveness of the model with respect to input features. Specifically, the method applies the fuzzy c-means (FCM) algorithm to historical data to uncover latent structural patterns via fuzzy partitioning. FCM allows each sample to simultaneously possess a membership degree to each of multiple cluster centers, thus effectively handling the ambiguity and uncertainty of input attributes in practical applications [42]. By calculating the membership degree of each sample to all cluster centers, the samples are divided into multiple categories, and the cluster centers and membership degrees are continuously optimized to obtain clustering results that meet expectations. The final cluster centers obtained are treated as candidate reference values, representing typical states of the input variables.

To identify the number of clusters (i.e., the number of reference values), the Davies–Bouldin index (DBI) is introduced as the evaluation criterion. This indicator measures the clustering quality by measuring the degree of dispersion (compactness) of samples within a cluster and the distance (separation) between center points between clusters. The smaller the DBI value is, the better the clustering effect is. By comparing DBIs across different cluster numbers, the number of clusters that minimize the DBI is chosen as the final reference value quantity, thus achieving a balance between model complexity and expressive power.

The reference values determined through this approach offer the following advantages: First, they align closely with the actual data distribution, thereby improving the model’s responsiveness to various input conditions. Second, the method eliminates the subjectivity introduced by manual specification, enhancing the objectivity and reproducibility of the modeling process. Third, by adaptively controlling the number of reference values within a reasonable range, the approach helps maintain the simplicity and interpretability of the BRB model. Overall, this data-driven reference value initialization strategy improves the modeling performance of BRB models and lays a solid foundation for accurate and reliable inference in uncertain and incomplete information. The following outlines the specific steps for reference value initialization using the DBI-FCM approach:

Step 1: Objective function definition

In BRB fault diagnosis, the quantity of reference values directly influences the quantity of rules and overall model complexity. To ensure model manageability, the quantity of reference values should be limited to a reasonable range. Since the number of rules grows exponentially with the number of reference values, having too many reference values leads to a dramatic increase in computational burden and difficulty in interpreting the model. Hence, appropriately limiting the number of reference values aids in both model optimization and enhancing user comprehension of the decision process.

According to cognitive psychology, human information processing capacity is inherently limited. Miller’s classic theory (i.e., “The Magical Number 7 ± 2”) suggests that humans can effectively handle only 5–9 discrete information units at a time [43]. This psychological constraint provides theoretical guidance for reference value selection: it is necessary to maintain a level of complexity that remains cognitively manageable. The boundary reference values in the BRB model usually match the dataset’s minimum and maximum values. Accordingly, this study limits the number of clusters (i.e., reference values) in the FCM process to the range of 2–7, ensuring that the resulting rule base retains adequate coverage while remaining interpretable and computationally tractable.

After determining the feasible range of cluster numbers, an objective function is formulated to direct the clustering process. This function aims to obtain the total Euclidean distances from all sample points to their corresponding cluster center. Through iterative optimization, the clustering converges, and the resulting cluster centers are then adopted as the BRB model’s reference values. The objective function is defined as

(14) $$F_{d}=\sum_{i=1}^{S}\sum_{k=1}^{H}v_{ik}^{d}{\left\|x_{i}-h_{k}\right\|}^{2},2\leq H\leq 7$$

where d is the fuzzification coefficient, which regulates the degree of cluster fuzziness. S represents the total number of samples. H represents the quantity of cluster centers. $h_{k}$ is the *k*th cluster center, sharing the same dimensionality as the input features. $x_{i}$ is the *i*th sample. $v_{ik}$ represents the membership degree of sample $x_{i}$ to cluster k. The Euclidean norm $\left\|*\right\|$ is calculated as

(15) $$m={\left\|x\right\|}^{2}=\sqrt{\sum_{i}x_{i}^{2}}$$

where m represents the Euclidean distance from the sample point to the cluster center.

Step 2: Compute membership matrix and cluster centers

The membership matrix, sized N×H, represents the degree of association between each sample point and each cluster. The cluster centers $h_{k}$ for each group are identified to minimize the objective function, ensuring maximum intra-cluster similarity. The membership degree and cluster centers are updated as

(16) $$v_{ik}=\frac{1}{\sum_{j=1}^{H}{\left(\frac{\left\|x_{i}-h_{k}\right\|}{\left\|x_{i}-h_{j}\right\|}\right)}^{\frac{2}{m-1}}}$$

(17) $$h_{k}=\frac{\sum_{i=1}^{S}v_{ik}^{m}x_{i}}{\sum_{i=1}^{S}v_{ik}^{m}}$$

Step 3: Set termination condition

The FCM algorithm terminates when the maximum change in the membership matrix between two consecutive iterations falls below a specified threshold:

(18) $$\max_{ij}\left\{\left|v_{ij}^{\left(t+1\right)}-v_{ij}^{\left(t\right)}\right|\right\}<\tau$$

where τ is a small positive constant representing the convergence criterion. t is the iteration index.

Step 4: Compute the DBI

For different numbers of clusters, steps 1 to step 3 are iterated to determine the respective cluster centers; then the DBI is computed as follows:

(19) $$DBI=\frac{1}{H}\sum_{k=1}^{H}\underset{i\neq k}{\max}\left[\frac{\nu_{k}+\nu_{i}}{{\left\|h_{k}-h_{j}\right\|}^{2}}\right]$$

(20) $$\nu_{k}={\left(\frac{1}{n_{k}}\sum_{j=1}^{n_{k}}{\left|X_{j}-h_{k}\right|}^{2}\right)}^{\frac{1}{2}}$$

where $v_{k}$ represents the average distance between intra-class data and cluster centroids.${\left\|h_{k}-h_{j}\right\|}^{2}$ represents the distance between the *i*th cluster and *j*th cluster centroids. $X_{j}$ represents the *j*th data point in the *i*th cluster. $n_{k}$ is the amount of data in the *i*th cluster.

Step 5: Discussion on threshold values

To satisfy the modeling requirements of the BRB model, the reference values of each input attribute must cover the full range of observed values in the dataset. In addition to the cluster centers automatically generated by the DBI-FCM method, the boundary values of each attribute should be explicitly included to build a complete set of the reference value. Specifically, the final set of reference values for each attribute consists of three parts:

(1) The minimum reference value, corresponding to the minimum observed value of the attribute within the dataset.
(2) The maximum reference value, corresponding to the maximum observed value of the attribute within the dataset.
(3) The intermediate reference values, corresponding to the cluster centers obtained through DBI-FCM.

Remark: The proposed DI-BRB-R method primarily focuses on the optimization and improvement of the basic BRB model and is suitable for system modeling and fault diagnosis in low-dimensional feature spaces. When dealing with higher-dimensional or larger-scale signal feature sets, the size of the BRB may grow exponentially, resulting in a “rule base explosion” problem that affects the readability of the BRB model [44]. To address this issue, hierarchical BRB structures can be considered, wherein features are grouped to construct multiple BRBs, and information is propagated from lower levels to higher levels through a hierarchical framework for evaluation. This approach effectively mitigates the challenges posed by high-dimensional features and rule base explosion. This will be a key focus of future research, aimed at enhancing the applicability and scalability of the method in complex high-dimensional environments.

### 3.2. Belief Degrees’ Initialization

In the application of the BRB model to fault diagnosis of complex systems, the construction of the belief rule base typically depends on domain expert knowledge to establish the initial rule structure and parameter settings. Among these, the belief degree of outcome serves as a quantitative expression of the reliability of the rule output, playing a critical role in subsequent inference and decision-making processes. However, in certain real-world scenarios, expert knowledge may be difficult to obtain or lack consistency, resulting in significant uncertainty in the initial belief degree assignment, which in turn compromises model performance [38].

To solve this issue, this paper introduces a data-driven belief degree initialization strategy under the assumption that the distribution of output grades is known. This method integrates a Gaussian membership function with a Laplace smoothing (LS-GMF) mechanism, utilizing the correlation characteristics between input data and output grades to automatically estimate the belief degrees of the rules. Specifically, the Gaussian membership function provides a probabilistic description of input-space fuzziness, while Laplace smoothing techniques are used to correct estimation bias caused by sparse samples. This method aims to improve the inference effectiveness of the model and modeling performance under uncertain or incomplete data conditions with limited expert knowledge. Prior to initializing belief degrees, the antecedent reference values and the consequent reference values should be determined, and a posterior probability distribution matrix should be constructed. The antecedent reference values are initialized in Section 3.2. The consequent reference values are specified by experts in the field. The posterior probability distribution matrix is used to record the belief degree allocation of each rule across different outcome grades during the inference process. Through this estimation process based on a combination of probability and smoothing, the uncertainty impact during the initialization phase can be effectively handled, providing assurance for the accuracy of subsequent inference results. Accordingly, the detailed initialization procedure is as follows:

Step 1: Calculate the membership degrees corresponding to the reference values

The membership degree is applied to measure the degree to which the training data match the reference values. First, based on the antecedent reference values obtained in the previous section, all possible combinations are generated via the Cartesian product. Then, the membership degree of each reference value in every combination is computed using the prescribed formula. Similarly, the membership degrees of the consequent reference values are also computed through the corresponding formula. The membership degree is calculated as follows:

(21) $$p(x_{A},\sigma ,A)=e^{-\frac{{\left(x_{A}-A\right)}^{2}}{2\sigma^{2}}}$$

(22) $$p(x_{D},\sigma ,D)=e^{-\frac{{\left(x_{D}-D\right)}^{2}}{2\sigma^{2}}}$$

where the membership degree of the antecedent reference values is computed using Formula (20), whereas the membership degree of the consequent reference values is computed using Formula (21). σ indicates the standard deviation of the data distribution. $x_{A}$ represents the input data of the antecedent attributes. $x_{D}$ denotes the input data of the outcomes.

Step 2: The calculation of the integrated membership degree

The integrated membership degree is obtained by combining the membership degrees of both the antecedent and consequent reference values. The calculation is as follows:

(23) $$P=(p_{i})p_{D},i=1,\ldots n$$

where $p_{i}(i=1,\ldots ,n)$ indicates the membership degree corresponding to the n antecedent attribute reference values within the combination. $p_{D}$ indicates the membership degree of the corresponding consequent reference value. P indicates the integrated membership degree, which corresponds to the posterior probability required for initialization.

Step 3: Verification procedure

In this step, the computed posterior probabilities are validated. If the training sample quantity is inadequate, the posterior probability for certain outcome grades may become zero, resulting in zero belief degrees for the corresponding rules, which prevents their activation in the BRB model. To mitigate this issue, the Laplace smoothing method is adopted. The corrected posterior probability is computed as follows:

(24) $$sp=\frac{\sum_{i=1}^{S}P_{\left(i,j\right)}^{k}+1}{\sum_{j=1}^{N}\left(\sum_{i=1}^{S}P_{\left(i,j\right)}^{k}\right)+N}$$

where $P_{\left(i,j\right)}^{k}$ denotes the posterior probability associated with the *k*th combination across all training samples.

Step 4: Normalization processing

Steps 1–3 are iteratively executed to compute the posterior probabilities for all the combinations, thereby obtaining the complete posterior probability distribution. The results are then normalized and recorded in the constructed posterior probability distribution matrix, yielding the initial belief degree distribution of the BRB.

### 3.3. Reliability Guidelines

In the fault diagnosis of diesel engines, model reliability is essential for ensuring the trustworthiness of diagnostic results. Due to the complex and variable nature of real-world operating conditions, a diagnostic system must not only achieve high accuracy but also maintain stability and consistency throughout the optimization process. The BRB model has been widely applied to such tasks, owing to its robust capability in handling uncertainty and its transparent reasoning mechanism [28]. The initial belief degree of this article is obtained through data-driven methods using the DI-BRB-R model, which reflects the characteristics of real-world datasets and provides a scientific basis for subsequent optimization. However, due to the randomness involved in optimization algorithms, unconstrained optimization may lead to excessive deviations of belief degrees from initial values. Such deviations may cause certain belief rules to contradict the actual operating rules of the system, thereby weakening the credibility and applicability of the model. To address this issue, a group of reliability guidelines is introduced to regulate the structure and parameters of the model during optimization. These guidelines are designed to guarantee that the optimized model performs well while retaining logical rationality and stability in practical diagnostic scenarios.

Reliability guideline 1: The belief degree distribution must be reasonable, consistent with real-world applications, and within feasible bounds.

The relationships between evaluation indicators and diagnostic results in diesel engine systems can be expressed using belief rules. The rationality of the rule design directly affects both the interpretability and diagnostic effectiveness of the BRB model [45]. However, in unconstrained optimization processes, certain belief rules may contradict the actual operational patterns of the system, thereby undermining the credibility and applicability of the model. To ensure that diagnostic outcomes remain consistent with the actual health condition of the diesel engine, it is essential to impose constraints on the belief degree distribution, thus constructing a group of belief rules that align with real-world semantics. The distribution of belief degrees should meet the following guideline:

(25) $$\begin{array}{l} \beta_{i,1}\leq \beta_{i,2}\leq \ldots \leq \beta_{i,N},\beta_{i,1}\geq \beta_{i,2}\geq \ldots \geq \beta_{i,N}\\ \beta_{i,1}\leq \ldots \leq \max(\beta_{i,1},\beta_{i,2},\ldots ,\beta_{i,N})\geq \ldots \geq \beta_{i,N}\\ \left(i=1,2,\ldots ,L,N=1,2,\ldots ,N\right) \end{array}$$

where $\beta_{i,N}$ represents the belief degree of the *N*th result of the *i*th rule.

To ensure the reliability of the model during the optimization process, the domain experts set reasonable constraint ranges for belief degrees during the optimization process based on the initial belief distribution of the DI-BRB-R model, namely the upper and lower limits of belief degrees. This constraint mechanism ensures that parameter adjustments during the optimization process are consistent with the initial belief degree distribution, improving the reliability of the model’s diagnostic results. Specifically, the belief degree of each rule must lie within a valid interval. The belief degree constraint is formulated as follows:

(26) $$\beta_{lb_{n,k}}\leq \beta_{n,k}\leq \beta_{ub_{n,k}}(n=1,2,\ldots ,N,k=1,2,\ldots ,L)$$

where $\beta_{ub_{n,k}}$ and $\beta_{lb_{n,k}}$ denote the upper and lower bounds of the belief degree $\beta_{n,k}$, respectively.

Reliability guideline 2: Rule weights and attribute weights should be optimized within feasible ranges.

The rule weights and attribute weights play a pivotal role in the inference process of BRB models, as they directly determine the relative contribution of individual rules and attributes to the final decision. Previous research has shown that unreasonable adjustment of weight distributions may diminish the influence of critical rules on decision outcomes, thereby introducing bias into the reasoning process and compromising the credibility of the model’s outputs. For instance, Cao et al. [41] found that rules and attributes assigned higher weights significantly affect the final decision. Therefore, to address this issue and maintain both structural rationality and logical consistency in inference, it is crucial to impose reasonable constraints on weight values during optimization. The following feasible ranges are imposed on the rule weights and attribute weights:

(27) $$\theta_{lb_{k}}\leq \theta_{k}\leq \theta_{ub_{k}}(k=1,2,\ldots ,L)$$

(28) $$\delta_{lb_{i}}\leq \delta_{i}\leq \delta_{ub_{i}}(i=1,2,\ldots ,T)$$

where $\theta_{lb}$ and $\theta_{ub}$ represent the upper and lower bounds of the rule weights, respectively. $\delta_{lb}$ and $\delta_{ub}$ denote the upper and lower bounds of the attribute weights, respectively.

### 3.4. The Optimization Process of the DI-BRB-R Model

To further enhance the accuracy of fault diagnosis, the projection covariance matrix adaptation evolution strategy (P-CMA-ES) is utilized to optimize the attribute weights, rule weights, and belief degrees of the DI-BRB-R model [46]. This method features an adaptive search strategy that dynamically responds to the characteristics of different optimization problems. Nevertheless, the original P-CMA-ES algorithm relies on global stochastic search, iteratively approaching the optimal solution through scattered sampling. While this process exhibits strong global search capabilities, it may lead to significant deviations of parameters from the initial values generated by the DI-BRB-R model, thereby compromising the model’s reliability. To address this issue, a group of reliability guidelines is introduced in Section 3.3 to improve the P-CMA-ES algorithm. In the improved P-CMA-ES optimization process, reliability constraints are applied after the initial population generation of each generation. The process first limits all parameters within the preset reliability constraint range to avoid values that exceed the constraint boundary. Then, the projection operation of equality constraints is performed on the relevant parameters belonging to the same group to meet the expected characteristics, thereby ensuring the rationality of the parameter distribution within the group. Thus, the improved P-CMA-ES can preserve optimization performance while effectively constraining parameter deviations, ensuring structural rationality during optimization and consistency with the initial parameters provided by the model.

An objective function is designed for the DI-BRB-R model to minimize the mean squared error (MSE) between actual outputs and predicted outputs under the given constraint conditions. The error is calculated as follows:

(29) $$MSE=\frac{1}{T}\sum_{i=1}^{T}\left(y_{i}^{actual}-y_{i}^{estimate}\right)$$

where $y_{i}^{actual}$ represents the actual result value. $y_{i}^{estimate}$ denotes the predicted value. T represents the amount of data.

According to the reliability guideline, the objective function under reliability constraints is defined as

(30) $$\begin{array}{l} \min\;MSE(\beta_{n,k},\theta_{k},\delta_{i})\\ s.t.\beta_{lb_{n,k}}\leq \beta_{n,k}\leq \beta_{ub_{n,k}},\\ \theta_{lb_{k}}\leq \theta_{k}\leq \theta_{ub_{k}},\\ \delta_{lb_{i}}\leq \delta_{i}\leq \delta_{ub_{i}},\\ \sum_{n=1}^{N}\beta_{n,k}\leq 1\\ \left(n=1,2,\ldots ,N,k=1,2,\ldots ,L,i=1,2,\ldots ,T\right) \end{array}$$

where the model accuracy is quantified using the MSE.

**Figure 1:**
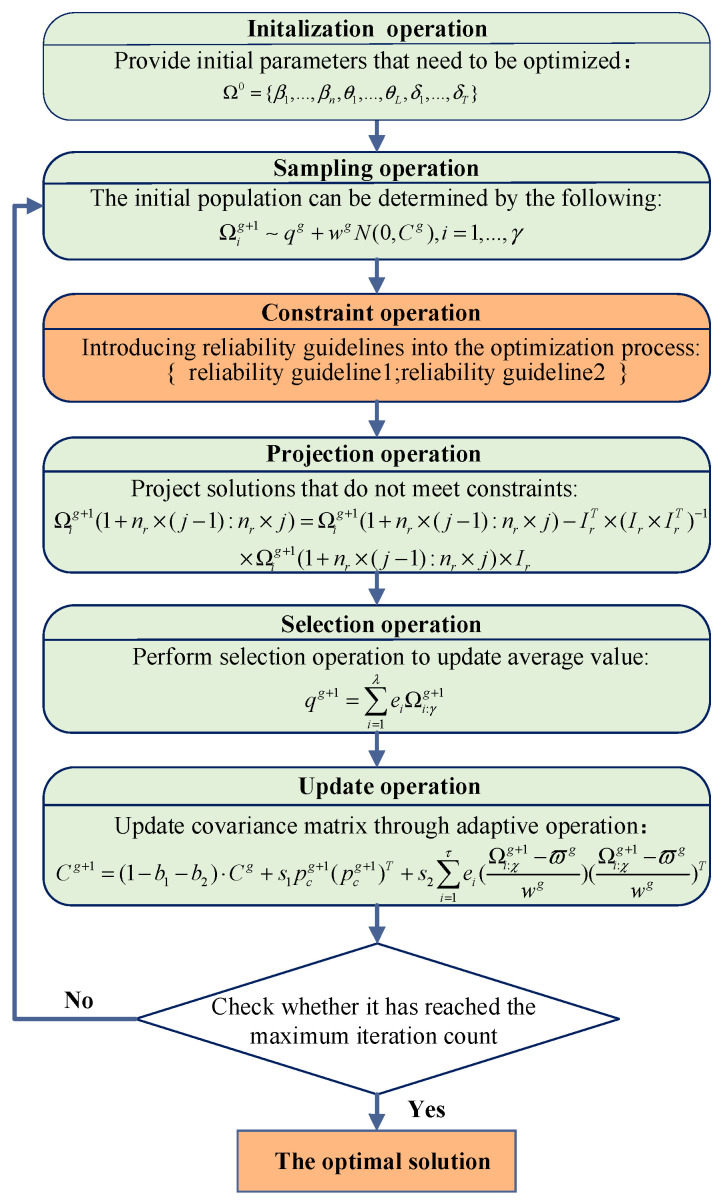
The specific optimization process of the improved P-CMA-ES.

The detailed optimization process of the improved P-CMA-ES method is illustrated in Figure 1, where $\Omega^{0}$ represents the initial belief degree obtained through DI-BRB-R and the initial attribute weights and the rule weights provided by experts. γ represents the population size. q represents the mean of the search distribution. w represents the step size. N(⋅) represents normal distribution. $C^{g}$ represents the covariance matrix of the *g*th generation. $\Omega_{i}^{g+1}$ refers to the solution of the *g*th generation. $n_{r}$ indicates the quantity of equality-constrained variables. $e_{i}$ denotes the weighting coefficient of the *i*th equation. $I_{r}$ denotes the parameter vector. $\Omega_{i:\gamma }^{g+1}$ indicates the *i*th solution of the *g*th generation. λ indicates the size of the offspring population. $b_{1},b_{2}$ denotes the learning rate. $p_{c}^{g+1}$ is the evolution path for the covariance matrix. $\vartheta^{g}$ denotes the generation step. $\varpi^{g}$ indicates the representative of the offspring population.

### 3.5. Summary of the DI-BRB-R Modeling

**Figure 2:**
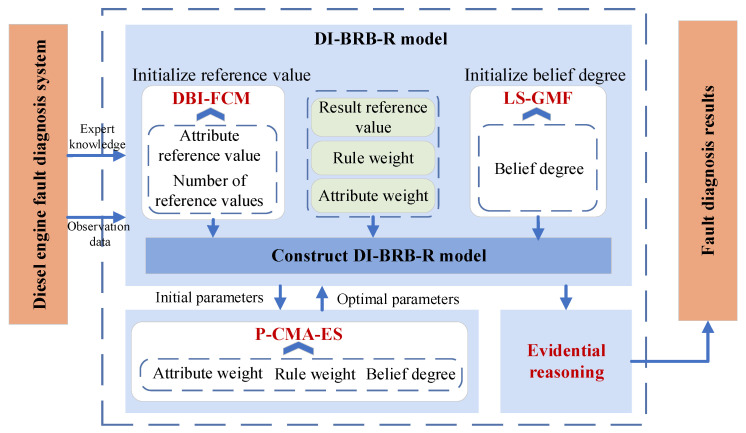
DI-BRB-R model for diesel engine fault diagnosis.

This section summarizes the modeling procedure of the DI-BRB-R model. Firstly, a data-driven approach is used to initialize the reference values and belief degrees required for constructing the DI-BRB-R model based on observation data. Then reliability constraints are added to the belief degrees of the model based on domain experts. Secondly, the improved P-CMA-ES optimization algorithm is used to optimize rule weights, attribute weights, and belief degrees. Finally, when inputting the observation data into the DI-BRB-R model, based on optimized parameters, the ER algorithm is used for fault diagnosis of the diesel engine to obtain the final diagnosis result, which is the state of the diesel engine. The overall modeling framework is illustrated in Figure 2, and the specific steps are as follows:

Step 1: Initialize the antecedent reference values using the DBI-FCM algorithm. The detailed procedure is described in Section 3.1.
Step 2: Initialize the belief degrees using a Gaussian membership function, as detailed in Section 3.2.
Step 3: Build the DI-BRB-R model using the reference values and belief degrees obtained in Step 1 and Step 2.
Step 4: Optimize the rule weights, attribute weights, and belief degrees through the improved P-CMA-ES algorithm. To prevent excessive deviation from the initial parameters provided by the DI-BRB-R model and damaging the reliability of the model, a group of reliability guidelines is introduced in Section 3.3 to guarantee the reliability of the optimization process.
Step 5: Based on the optimized parameters, the ER algorithm is employed to derive the final fault diagnosis results for the diesel engine.

## 4. Case Study

In this section, we describe how the effectiveness of the DI-BRB-R model was validated through fault diagnosis of the WD615 diesel engine system.

In this experiment, the WD615 diesel engine was selected as the test subject. Vibration sensors were installed on the left side of the engine cylinder and the oil sump baffle, and the clearance between the piston and cylinder was adjusted to three different values to simulate normal operation, moderate fault, and severe fault conditions, respectively [47]. The engine’s steady-state speed was then set to 1800 r/pm, with a sampling frequency of 12.8 kHz, and the corresponding vibration signals were collected from the sensors. According to fault characteristics, the diesel engine’s fault conditions were categorized into three classes: normal (N), moderate fault (M), and severe fault (S), with the corresponding reference values presented in Table 1 [48].

### 4.1. The Modeling of the DI-BRB-R

The vibration signals were collected under each operational state. The mean and kurtosis were extracted as primary observation indicators using the time-domain feature extraction method from reference [47]. In time-domain feature analysis, the mean and kurtosis are significant statistical measures reflecting signal characteristics:

(1) Mean: The mean indicates the average amplitude of the signal during a specified time interval, reflecting its overall energy level:

(31) Mean=mean[x(n)]

(2) Kurtosis: Kurtosis measures the distribution characteristics of signal amplitudes around the mean, which can be used to identify outliers or indicate the sharpness of the signal:

(32) $$Kurtosis=mean[{\left(x(n)-\bar{x}\right)}^{4}/\sigma^{4}]$$

**Figure 3:**
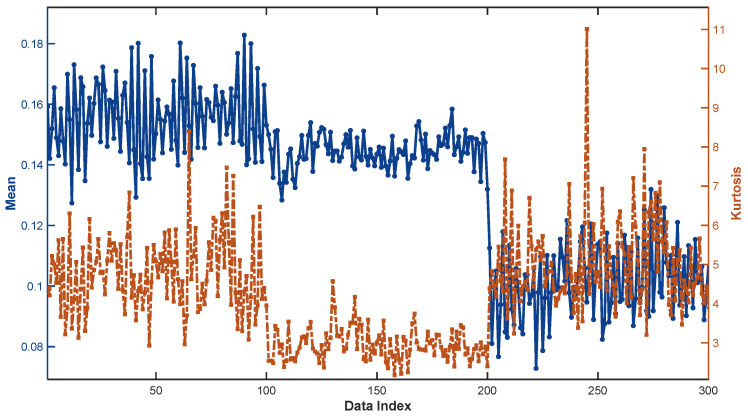
Data distribution diagram of two attributes of WD615 diesel engine.

In the data processing procedure, one feature set was extracted from every 15 groups of raw vibration data, resulting in a total of 300 feature samples [36]. Among these, there were 100 samples each for the severe fault, moderate fault, and normal states. Of the total, 150 samples were applied as training data and the remaining 150 as testing data. The data distribution is illustrated in Figure 3. The maximum and minimum values of the mean were 0.1829 and 0.0728, respectively, while those of kurtosis were 11.0112 and 2.1749, which served as the border values for the reference values of the mean and kurtosis. The DBI-FCM method described in Section 3.1 was utilized to determine the other reference values.

In this experiment, a data-driven initialization was performed for the reference values of the antecedent mean and kurtosis attributes using the DBI-FCM method. As specified in Formula (14), the search range for the number of clusters was set from 2 to 7. The FCM algorithm was then applied to obtain the cluster centers for each specified cluster count. To assess the quality of each clustering result, the corresponding DBI was calculated, which served as a criterion for assessing clustering performance and guiding the selection of the optimal quantity of clusters. The cluster centers and DBI obtained for the mean and kurtosis attributes under different clustering conditions are summarized in Table 2 and Table 3. According to Table 2 and Table 3, the best DBI for both the mean and kurtosis were achieved when the quantity of clusters was set to 2. Therefore, the additional reference values for the mean were determined to be 0.0993 and 0.1494, and those for Kurtosis were 3.2291 and 5.2068. The final reference values, attribute weights, and reliability constraint intervals defined for attribute weights based on the reliability guideline 2 in Section 3.3 are listed in Table 4, where their initial attribute weights all set to 1. Based on the Cartesian product of the reference values, a total of 16 rules are generated. As each rule corresponds to three fault states, the model requires 16 × 3 = 48 belief degrees in total.

The belief degrees were initialized using the LS-GMF method described in Section 3.2. To verify the effectiveness and appropriateness of the selected standard deviations for the Gaussian membership function, a comprehensive sensitivity analysis was conducted to examine the influence of varying standard deviation values on model accuracy. The optimal standard deviations were determined to be 0.02 for the mean, 0.7 for kurtosis, and 0.4 for the fault states. These values were chosen through a quantitative assessment of their influence on membership degree variation and the model’s overall performance. This ensured that sample points closer to the distribution centroid received higher membership degrees, thus capturing the local features of the data more effectively. Subsequently, Formulas (20) and (21) were applied to compute the membership degrees of the input sample relative to each antecedent reference value and each fault state, respectively. Based on these membership degrees, Formula (22) was used to compute the corresponding integrated membership degrees. Each rule was then examined to determine whether Laplace smoothing correction was required. Finally, the initial belief distribution of the BRB was obtained by normalizing the integrated membership degrees from all inputs and recording them in the constructed posterior probability distribution matrix. Table 5 shows the initial belief rule base of the DI-BRB-R model, as well as the reliability constraints defined for the belief rule base and rule weights based on the reliability guidelines in Section 3.3, where all rule weights are initialized to 1.

**Figure 4:**
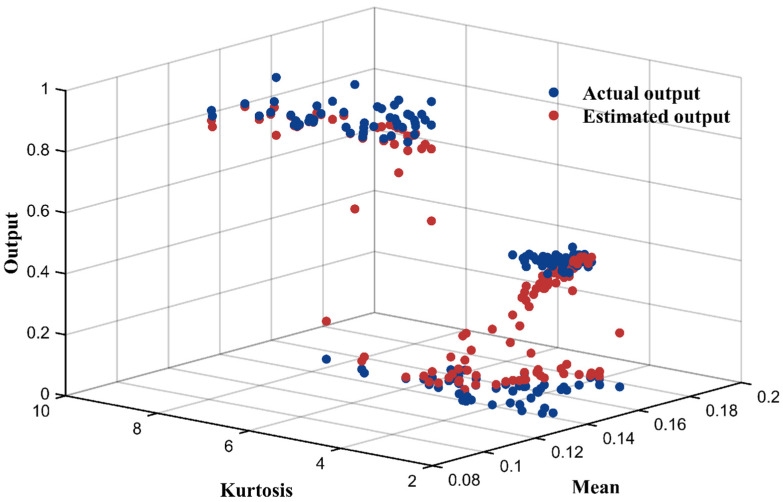
Comparison between fault diagnosis output and actual value of DI-BRB-R.

The model parameters, including rule weights, attribute weights, and belief degrees, were further optimized through the improved P-CMA-ES algorithm to improve model accuracy. The optimization process was run for 200 iterations. Table 6 presents the optimized belief rule base, and Table 7 lists the optimized attribute weights. The test result yielded an MSE of 0.0122, and the prediction performance is illustrated in Figure 4.

### 4.2. Analysis of the Experimental Results

In this paper, DI-BRB-R is the proposed model, and its initial parameters were optimized by an improved P-CMA-ES algorithm. DI-BRB refers to the DI-BRB-R model optimized using the traditional P-CMA-ES method (i.e., the P-CMA-ES optimization method without the reliability criterion). DI-BRB-0 refers to the DI-BRB-R model without any optimization method.

**Figure 5:**
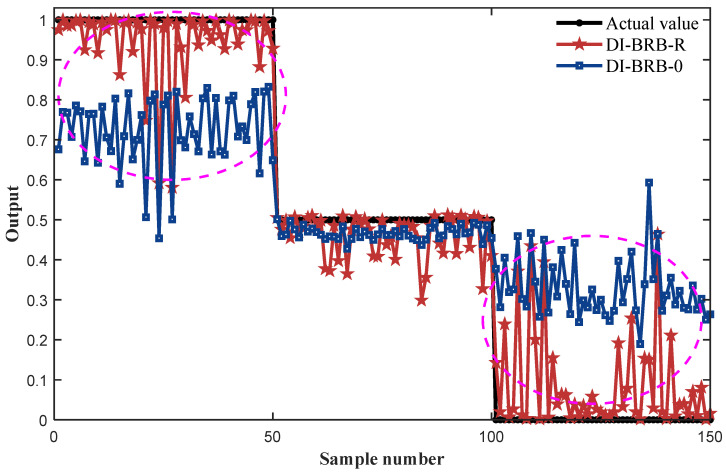
Fault diagnosis results of two types of DI-BRB-R.

In Section 4.1, a diesel engine fault diagnosis model based on the DI-BRB-R is established. The MSE of DI-BRB-0 is 0.0662. DI-BRB-R achieved an MSE of 0.0120 in testing. Figure 5 presents a comparative analysis of the diagnostic results of DI-BRB-0, the DI-BRB-R model, and the actual fault states. The results demonstrate that the fault diagnosis model constructed by the DI-BRB-R model not only achieves high accuracy in identifying diesel engine faults but also effectively improves the modeling ability of the BRB by introducing data-driven methods. The part circled by the dashed circle clearly indicates that the DI-BRB-R model can better and accurately diagnose faults in diesel engines. Consequently, even when expert knowledge is limited, the proposed DI-BRB-R model remains effective in diagnosing the fault states of diesel engines.

### 4.3. The Reliability Analysis of the Model

**Figure 6:**
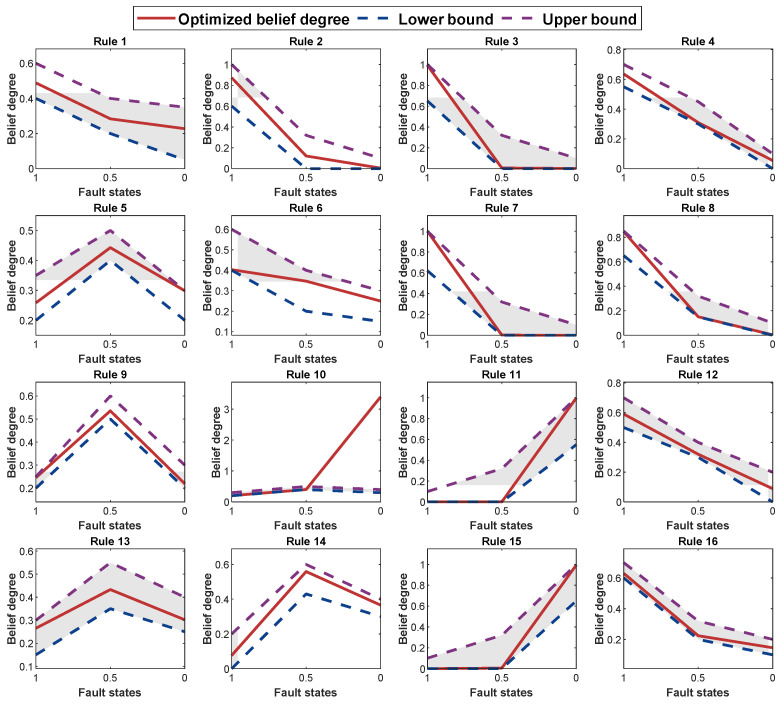
The belief distribution of each rule in the optimized DI-BRB-R.

**Figure 7:**
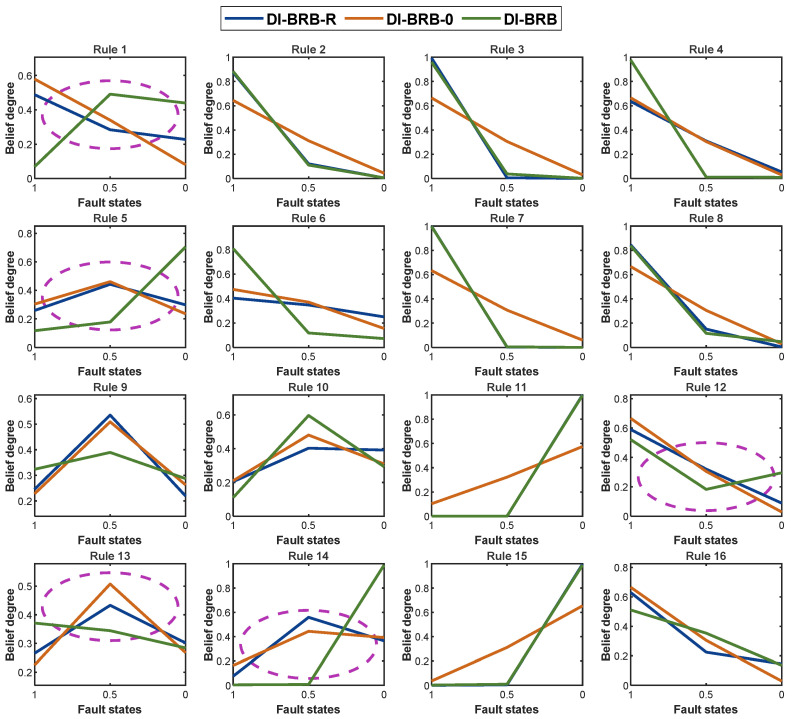
Comparison of belief distribution between DI-BRB-R and other methods.

To preserve the initial DI-BRB-R model’s reliability, this study introduces a group of reliability guidelines in Section 3.3 to guide the P-CMA-ES optimization method. By imposing these reliability constraints during the P-CMA-ES optimization process, the model improves accuracy while ensuring the reliability of the DI-BRB-R model. As shown in Figure 6, all belief degrees in the DI-BRB-R model are effectively constrained within the reliable intervals defined according to reliability guideline 1. Figure 7 presents a comparison of the belief degrees for each rule across three models: DI-BRB-R, DI-BRB-0, and DI-BRB. As shown in Figure 7, the belief degrees obtained by DI-BRB deviate from those of DI-BRB-0. In particular, the rule belief degrees highlighted by the circle in Figure 7 exhibit substantial deviations from those of DI-BRB-0. For example, in Rule 5, while the belief distribution from DI-BRB-0 is originally monotonic, DI-BRB produces a convex distribution, altering the original shape. This deviation undermines the reliability of the DI-BRB-0 model. In contrast, the belief degrees obtained by DI-BRB-R remain consistent with those of DI-BRB-0. These results clearly demonstrate that introducing the reliability constraint effectively ensures that the optimized belief distribution maintains its consistency with the original distribution. Therefore, the proposed DI-BRB-R model exhibits higher reliability.

### 4.4. Comparative Experiment

**Figure 8:**
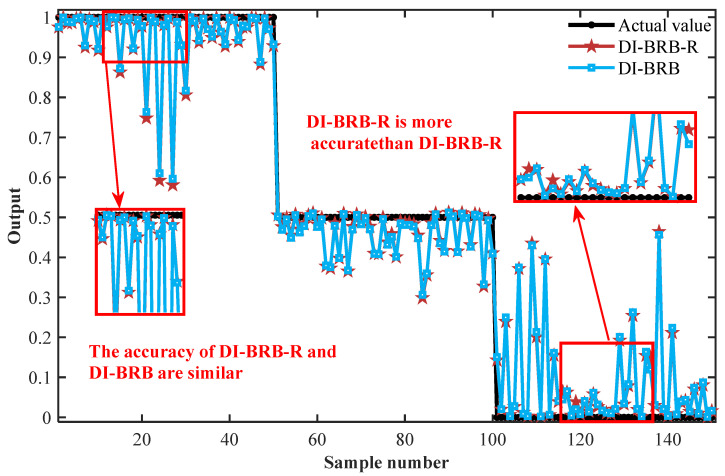
Comparison of diagnosis results between DI-BRB-R and DI-BRB.

To further validate the performance of the DI-BRB-R model, this section compares its fault diagnosis results with those of other BRB models and advanced diagnostic methods. The BRB models considered include the classical BRB algorithm, BRB models proposed by Feng et al. [48], Li et al. [36], and Yin et al. [32]. The advanced models include a backpropagation neural network (BPNN), support vector machine (SVM), extreme learning machine (ELM), and random forest (RF). All the comparison models mentioned above use the same input features (mean and kurtosis) to diagnose faults in diesel engines on the same training and testing datasets (150 training samples, 150 testing samples). The BPNN, SVM, ELM, and RF all underwent detailed hyperparameter tuning to achieve their respective optimal performance. The comparative study consisted of two parts, and Table 8 presents the relevant comparative results (averaged over 50 independent trials) from three performance indicators: the MSE, root mean squared error (RMSE), and mean absolute error (MAE). In the first part, the DI-BRB-R model performed slightly less accurately than DI-BRB, mainly due to the influence of the reliability constraints. Figure 8 shows a comparison of the fault diagnosis results between DI-BRB-R and DI-BRB. Under “normal conditions,” both models exhibited similar diagnostic accuracy. Under “severe fault” conditions, DI-BRB achieved slightly higher accuracy than DI-BRB-R. Therefore, under reliability constraints, the DI-BRB-R model will not significantly impact the accuracy of fault diagnosis. In addition, compared with the three BRB models proposed by Feng, Li, Yin, and others, the DI-BRB-R model exhibits significant accuracy advantages. These three models are based on BRBs constructed with sufficient domain expert knowledge, which can achieve good diagnostic results in scenarios with sufficient expert knowledge. However, when expert knowledge is insufficient, traditional methods may have certain limitations in model construction, leading to a decrease in model performance. In contrast, the DI-BRB-R model employs a data-driven approach to fully extract historical data features for initializing the reference values and belief degrees required in BRB construction. This approach reduces reliance on expert knowledge, compensates for its insufficiency, and simultaneously improves diagnostic accuracy while ensuring model reliability. In the second part, the DI-BRB-R model achieved higher accuracy compared to other advanced models. Overall, the results demonstrate that DI-BRB-R exhibits strong capability in initial model construction by effectively integrating limited expert knowledge with data-driven approaches, while ensuring that the reliability of the original model is not compromised by the optimization process. This improvement enables the DI-BRB-R model to not only provide accurate diagnostic results but also provide a clear reasoning process, thereby enhancing users’ trust and understanding of the model output, making diagnostic results more reliable and easily accepted by users.

### 4.5. Experimental Summary

Diesel engines often operate in harsh environments, where input data may be affected by fuzziness and uncertainty, making it difficult or even impossible for experts to accurately obtain the parameters of reference values and belief degrees. Experimental results demonstrate that the DI-BRB-R model can still achieve reasonable initialization parameters and deliver excellent diagnostic performance under such conditions, providing a reliable and effective modeling approach for diesel engine fault diagnosis. In the early modeling stage, the model relies only on partial expert knowledge and effectively constructs the initial BRB model by incorporating a data-driven mechanism, significantly enhancing the modeling capability and adaptability. Meanwhile, the DI-BRB-R model introduces a set of reliability criteria to ensure that the model parameters evolve within a reasonable and reliable range during the P-CMA-ES optimization process, thereby improving optimization accuracy while maintaining model reliability. The experimental results in Table 8 further show that even in the face of uncertainty in diesel engine operation, the proposed method achieves diagnostic performance comparable to or better than existing methods, confirming its applicability under conditions of uncertainty or insufficient expert knowledge. This method provides robust technical support for diesel engine design optimization, performance improvement, and fault risk control and holds promising engineering application prospects as well as theoretical significance.

## 5. Conclusions

To address the issue of limited expert knowledge in diesel engine fault diagnosis, a fault diagnosis for diesel engine method based on the DI-BRB-R model is proposed in this paper. By incorporating a data-driven initialization approach to supplement the expert knowledge required for diagnosis, the model’s modeling capability is significantly enhanced. The experimental results demonstrate that the proposed method can analyze the fault states of diesel engines more accurately and comprehensively while maintaining model reliability. The main contributions of this work are as follows: (1) To address the challenge of insufficient expert knowledge in diesel engine fault diagnosis, the DI-BRB-R model is developed. (2) To ensure the effectiveness of the model, a fuzzy c-means clustering method with the Davies–Bouldin index (DBI-FCM) and a Gaussian membership function method with Laplacian smoothing (LS-GMF) are proposed, which are used to initialize attribute reference values and belief degrees, respectively, to improve the construction efficiency and prediction accuracy of the BRB model. (3) To prevent optimization algorithms from compromising the reliability of the initially constructed BRB model, a group of reliability guidelines is introduced to ensure the reliability of the model while improving its accuracy.

This approach not only enhances the modeling capability under conditions of limited expert knowledge but also broadens the applicability of the method in real-world industrial applications, such as condition monitoring and fault diagnosis of complex mechanical systems. Due to it effectively reducing the dependence on domain expertise and improving diagnostic reliability and accuracy, the method is well suited for engineering environments where data uncertainty and expert scarcity are common.

However, the model still has certain limitations. This method is more suitable for problems with low dimensional attributes, and the modeling process is relatively complex. For high-dimensional or multi-attribute problems, the DI-BRB-R model may face scalability challenges, potentially limiting its practicality in some large-scale systems. Future research will focus on these issues with the aim of further enhancing the adaptability and feasibility of industrial applications of this method.

## Figures and Tables

**Table 1:** The reference values of fault states.

Rank	N	M	S
Reference values	1	0.5	0

**Table 2:** Clustering results of mean attribute.

Number of Clusters	The Cluster Center Value	DBI
2	{0.0993, 0.1494}	0.8972
3	{0.0977, 0.1424, 0.1648}	3.1303
4	{0.0899, 0.1102, 0.1439, 0.1663}	2.5632
5	{0.0860, 0.1057, 0.1382, 0.1490, 0.1682}	4.3898
6	{0.0852, 0.1041, 0.1296, 0.1422, 0.1514, 0.1691}	5.0570
7	{0.0824, 0.0968, 0.1065, 0.1203, 0.1411, 0.1510, 0.1689}	5.1407

**Table 3:** Clustering results of kurtosis attribute.

Number of Clusters	The Cluster Center Value	DBI
2	{3.2291, 5.2068}	1
3	{3.0129, 4.5144,5.9645}	1.3033
4	{2.7662, 3.6161, 4.6544, 6.0063}	3.6062
5	{2.7224, 3.4963, 4.5043,5.5631, 7.1421}	3.0987
6	{2.7008, 3.4277,4.3236,4.8954, 5.6777, 7.1333}	8.2395
7	{2.6248,3.1666,3.6413, 4.3603, 4.9168, 5.6757, 7.0940}	5.6760

**Table 4:** Reference values for mean attribute.

Attribute	Attribute Weight	Mean1	Mean2	Mean3	Mean4
Mean	1 [0.9, 1]	0.0728	0.0993	0.1494	0.1829
Kurtosis	1 [0.9, 1]	2.1749	3.2291	5.2068	11.0112

**Table 5:** Initial belief rules table.

No.	Attribute		Rule Weight	$\mathrm{Initial}\;\mathrm{Belief}\;\mathrm{Degree}\;\{\beta_{1},\beta_{2},\beta_{3}\}$
	Mean	Kurtosis		
1	Mean1	Kurtosis1	1 [0.5, 1]	{0.5790, 0.3404, 0.0806} {[0.4, 0.6], [0.2, 0.4], [0.05, 0.35]}
2	Mean1	Kurtosis2	1 [0.6, 1]	{0.6440, 0.3113, 0.0447} {[0.6, 1], [0, 0.32], [0, 0.1]}
3	Mean1	Kurtosis3	1 [0.8, 1]	{0.6644, 0.3050, 0.0306} {[0.65, 1], [0, 0.32], [0, 0.1]}
4	Mean1	Kurtosis4	1 [0.7, 1]	{0.6659, 0.3049, 0.0293} {[0.55, 0.7], [0.3, 0.45], [0, 0.1]}
5	Mean2	Kurtosis1	1 [0.5, 1]	{0.3035, 0.4613, 0.2352} {[0.2, 0.35], [0.4, 0.5], [0.2, 0.3]}
6	Mean2	Kurtosis2	1 [0, 1]	{0.4745, 0.3703, 0.1552} {[0.4, 0.6], [0.2, 0.4], [0.15, 0.3]}
7	Mean2	Kurtosis3	1 [0.8, 1]	{0.6329, 0.3072, 0.0599} {[0.62, 1], [0, 0.32], [0, 0.1]}
8	Mean2	Kurtosis4	1 [0.8, 1]	{0.6659, 0.3049, 0.0293} {[0.65, 0.85], [0.15, 0.32], [0, 0.1]}
9	Mean3	Kurtosis1	1 [0.7, 1]	{0.2282, 0.5090, 0.2628} {[0.2, 0.25], [0.5, 0.6], [0.2, 0.3]}
10	Mean3	Kurtosis2	1 [0.2, 1]	{0.2098, 0.4803, 0.3099} {[0.2, 0.3], [0.4, 0.5], [0.3, 0.4]}
11	Mean3	Kurtosis3	1 [0.4, 1]	{0.1031, 0.3222, 0.5747} {[0, 0.1], [0, 0.32], [0.55, 1]}
12	Mean3	Kurtosis4	1 [0.1, 1]	{0.6659, 0.3049, 0.0293} {[0.5, 0.7], [0.3, 0.4], [0, 0.2]}
13	Mean4	Kurtosis1	1 [0.7, 1]	{0.2250, 0.5074, 0.2676} {[0.15, 0.3], [0.35, 0.55], [0.25, 0.4]}
14	Mean4	Kurtosis2	1 [0, 1]	{0.1641, 0.4438, 0.3921} {[0, 0.2], [0.43, 0.6], [0.3, 0.4]}
15	Mean4	Kurtosis3	1 [0.8, 1]	{0.0363, 0.3105, 0.6532} {[0, 0.1], [0, 0.32], [0.65, 0.1]}
16	Mean4	Kurtosis4	1 [0.6, 1]	{0.6659, 0.3049, 0.0293} {[0.6, 0.7], [0.2, 0.32], [0.1, 0.2]}

**Table 6:** Optimized belief rules table.

No.	Attribute		Rule Weight	$\mathrm{Optimized}\;\mathrm{Belief}\;\mathrm{Degree}\;\{\beta_{1},\beta_{2},\beta_{3}\}$
	Mean	Kurtosis		
1	Mean1	Kurtosis1	0.8126	{0.5158, 0.3473, 0.1369}
2	Mean1	Kurtosis2	0.7879	{0.9610, 0.0361, 0.0029}
3	Mean1	Kurtosis3	0.8802	{0.9991,0.0004, 0.0004}
4	Mean1	Kurtosis4	0.8599	{0.3849, 0.5915, 0.0235}
5	Mean2	Kurtosis1	0.5406	{0.1278, 0.4192, 0.4530}
6	Mean2	Kurtosis2	0	{0.5928, 0.1626, 0.2445}
7	Mean2	Kurtosis3	0.9863	{0.9993, 0.0002, 0}
8	Mean2	Kurtosis4	0.8129	{0.8866, 0.1047, 0.0087}
9	Mean3	Kurtosis1	0.9320	{0.2431, 0.5505, 0.2064}
10	Mean3	Kurtosis2	0.2756	{0.2339, 0.3406, 0.4255}
11	Mean3	Kurtosis3	0.5093	{0.0001, 0.0005, 0.9994}
12	Mean3	Kurtosis4	0.1692	{0.2582, 0.4633, 0.2780}
13	Mean4	Kurtosis1	0.9493	{0.1699, 0.3596, 0.4705}
14	Mean4	Kurtosis2	0	{0.0019, 0.0042, 0.9938}
15	Mean4	Kurtosis3	0.9931	{0.0019, 0.0042, 0.9938}
16	Mean4	Kurtosis4	0.6079	{0.6010, 0.3041, 0.0949}

**Table 7:** Optimized attribute weights.

Attribute	Attribute Weight
Mean	1
Kurtosis	0.9967

**Table 8:** Comparative study of DI-BRB-R with different models.

	Method	MSE	RMSE	MAE
Part A	Classical BRB	0.1560	0.3950	0.3491
	DI-BRB	0.0120	0.1097	0.0591
	BRB [Feng]	0.0398	0.1955	0.1471
	BRB [Li]	0.0287	0.1694	0.1039
	BRB [Yin]	0.0425	0.2062	0.1503
Part B	DI-BRB-R	0.0122	0.1103	0.0601
	BPNN	0.0228	0.1508	0.0933
	SVM	0.1345	0.3668	0.2942
	ELM	0.0421	0.2051	0.1026
	RF	0.0301	0.1735	0.1094

## Data Availability

Due to privacy concerns, the authors do not have permission to share data.

## References

[1] Yan H., Bai H., Zhan X., Wu Z., Wen L., Jia X. (2022). Combination of VMD Mapping MFCC and LSTM: A New Acoustic Fault Diagnosis Method of Diesel Engine. Sensors.

[2] Ming Z., Zhou Z., Cao Y., Tang S., Chen Y., Han X., He W. (2023). A new interpretable fault diagnosis method based on belief rule base probability table. Chin. J. Aeronaut..

[3] Zhou Z., Ming Z., Wang J., Tang S., Cao Y., Han X., Xiang G. (2023). A Novel Belief Rule-Based Fault Diagnosis Method with Interpretability. Comput. Model. Eng. Sci..

[4] Zhou F., Yang S., Fujita H., Chen D., Wen C. (2020). Deep learning fault diagnosis method based on global optimization GAN for unbalanced data. Knowl.-Based Syst..

[5] Cai B., Sun X., Wang J., Yang C., Wang Z., Kong X., Liu Z., Liu Y. (2020). Fault detection diagnostic method of diesel engine by combining rule-based algorithm BNs/BPNNs. J. Manuf. Syst..

[6] Deng Q., Li S., Yang C., Ma N., He W. (2024). A New Health State Assessment Method for Complex Systems Based on Approximate Belief Rule Base With Attribute Reliability. IEEE Access.

[7] Li S., Qi L., Shi J., Xiao H., Da B., Tang R., Zuo D. (2024). Study on Few-Shot Fault Diagnosis Method for Marine Fuel Systems Based on DT-SViT-KNN. Sensors.

[8] Kong X., Cai B., Khan J.A., Gao L., Yang J., Wang B., Yu Y., Liu Y. (2024). Concurrent fault diagnosis method for electric-hydraulic system: Subsea blowout preventer system as a case study. Ocean Eng..

[9] Qi J., Mauricio A., Sarrazin M., Janssens K., Gryllias K. (2018). Enhanced Particle Filter and Cyclic Spectral Coherence based Prognostics of Rolling Element Bearings. PHM Soc. Eur. Conf..

[10] Gao B., Xu J., Zhang Z., Liu Y., Chang X. (2024). Marine diesel engine piston ring fault diagnosis based on LSTM, improved beluga whale optimization. Alex. Eng. J..

[11] Li W., Liu X., Wang D., Lu W., Yuan B., Qin C., Cheng Y., Căleanu C. (2024). MITDCNN: A multi-modal input Transformer-based deep convolutional neural network for misfire signal detection in high-noise diesel engines. Expert Syst. Appl..

[12] Wang R., Yan H., Dong E., Cheng Z., Li Y., Jia X. (2024). Infrared thermography based fault diagnosis of diesel engines using convolutional neural network and image enhancement. Open Phys..

[13] Yang X., Bi F., Cheng J., Tang D., Shen P., Bi X. (2024). A Multiple Attention Convolutional Neural Networks for Diesel Engine Fault Diagnosis. Sensors.

[14] Chen T., Xiang Y., Wang X. (2025). IFD-BiC: A class-incremental continual learning method for diesel engine fault diagnosis. Eng. Res. Express.

[15] Yang L., Fu W., Li W., Li H., Xu R., Zhang S. (2025). Response and fault diagnosis of crankshafts containing breathing cracks based on torsional angular velocity. Mech. Syst. Signal Process..

[16] Xu N., Yang L., Guo Y., Chang L., Zhang G., Zhang J. (2025). An Improved Thermoeconomic Diagnosis Method: Applying to Marine Diesel Engines. J. Mar. Sci. Eng..

[17] Coelho R.N.C., Macêdo E.N., Quaresma J.N.N. (2023). Monitoring the operational condition of a diesel engine by evaluating the parameters of its thermodynamic operation cycle. J. Braz. Soc. Mech. Sci. Eng..

[18] Knežević V., Orović J., Stazić L., Čulin J. (2020). Fault Tree Analysis Failure Diagnosis of Marine Diesel Engine Turbocharger System. J. Mar. Sci. Eng..

[19] Du J., Ma K., Liu Y. (2024). Research on the dynamic characteristics of multi-cylinder crankshaft considering crack and engine variable conditions. Nonlinear Dyn..

[20] Zhan X., Bai H., Yan H., Wang R., Guo C., Jia X. (2022). Diesel Engine Fault Diagnosis Method Based on Optimized VMD, Improved, CNN. Processes.

[21] Guo Y., Zhang J. (2023). Fault Diagnosis of Marine Diesel Engines under Partial Set Cross Working Conditions Based on Transfer Learning. J. Mar. Sci. Eng..

[22] Jiang R., Ou S., Li B., Liu W., Cao B., Yu Y., Katunin A. (2025). A Fault Diagnosis Method for Typical Failures of Marine Diesel Engines Based on Multisource Information Fusion. Shock Vib..

[23] Li B., Ding Y., Ma W., Xiang L., Sui C. (2025). A health condition assessment method for marine diesel engine turbochargers using zero-dimensional engine model and machine learning. Measurement.

[24] Li H., Liu F., Kong X., Zhang J., Jiang Z., Mao Z. (2023). Knowledge features enhanced intelligent fault detection with progressive adaptive sparse attention learning for high-power diesel engine. Meas. Sci. Technol..

[25] Qi J., Chen Z., Uhlmann Y., Schullerus G. (2025). Sensorless Robust Anomaly Detection of Roller Chain Systems Based on Motor Driver Data Deep Weighted, K.N.N. IEEE Trans. Instrum. Meas..

[26] Kong X., Cai B., Yu Y., Yang J., Wang B., Liu Z., Shao X., Yang C. (2025). Intelligent diagnosis method for early faults of electric-hydraulic control system based on residual analysis. Reliab. Eng. Syst. Saf..

[27] Qi J., Chen Z., Kong Y., Qin W., Qin Y. (2025). Attention-guided graph isomorphism learning: A multi-task framework for fault diagnosis and remaining useful life prediction. Reliab. Eng. Syst. Saf..

[28] Zhou Z., Hu G., Hu C., Wen C., Chang L. (2021). A Survey of Belief Rule-Base Expert System. IEEE Trans. Syst. Man Cybern. Syst..

[29] Yang J., Liu J., Wang J., Sii H., Wang H. (2006). Belief rule-base inference methodology using the evidential reasoning, Approach-RIMER. IEEE Trans. Syst. Man Cybern.-Part A Syst. Hum..

[30] Chang L., Zhang L., Fu C., Chen Y.W. (2022). Transparent Digital Twin for Output Control Using Belief Rule Base. IEEE Trans. Cybern..

[31] Zhang Y., Du Y., He W., Zhang L., Wu R. (2025). A new belief rule base model with uncertainty parameters. Reliab. Eng. Syst. Saf..

[32] Yin X., He W., Cao Y., Zhou G., Li H. (2023). Interpretable belief rule base for safety state assessment with reverse causal inference. Inf. Sci..

[33] Liu M., He W., Ma N., Zhu H., Zhou G. (2025). A new reliability health status assessment model for complex systems based on belief rule base. Reliab. Eng. Syst. Saf..

[34] Chang L., Xu X., Liu Z., Qian B., Xu X., Chen Y. (2021). BRB Prediction With Customized Attributes Weights Tradeoff Analysis for Concurrent Fault Diagnosis. IEEE Syst. J..

[35] Xu X., Yan X., Sheng C., Yuan C., Xu D., Yang J. (2020). A Belief Rule-Based Expert System for Fault Diagnosis of Marine Diesel Engines. IEEE Trans. Syst. Man Cybern. Syst..

[36] Li H., Yin X., He W., Feng Z., Cao Y. (2023). A New Fault Diagnosis Method Based on Belief Rule Base With Attribute Reliability Considering Multi-Fault Features. IEEE Access.

[37] Zhang Q., Si Z., Shen J., Zhu H., Zhou G., He W. (2025). Data-driven enhanced belief rule base for complex system health state assessment. Inf. Sci..

[38] Zhang Q., Zhao B., He W., Zhu H., Zhou G. (2024). A behavior prediction method for complex system based on belief rule base with structural adaptive. Appl. Soft Comput..

[39] Wu J., Wang Q., Wang Z., Zhou Z. (2022). AutoBRB: An automated belief rule base model for pathologic complete response prediction in gastric cancer. Comput. Biol. Med..

[40] Yang J., Xu D. (2013). Evidential reasoning rule for evidence combination. Artif. Intell..

[41] Cao Y., Zhou Z., Hu C., He W., Tang S. (2021). On the Interpretability of Belief Rule-Based Expert Systems. IEEE Trans. Fuzzy Syst..

[42] Tang M., Liao H., Xu J., Streimikiene D., Zheng X. (2020). Adaptive consensus reaching process with hybrid strategies for large-scale group decision making. Eur. J. Oper. Res..

[43] Miller G.A. (1956). The magical number seven, plus or minus two: Some limits on our capacity for processing information. Psychol. Rev..

[44] Cao Y., Tang S., Yao R., Chang L., Yin X. (2024). Interpretable hierarchical belief rule base expert system for complex system modeling. Measurement.

[45] Si Z., Shen J., He W. (2024). Lithium-Ion Battery Health Assessment Method Based on Double Optimization Belief Rule Base with Interpretability. Batteries.

[46] Zhou Z., Hu G., Zhang B., Hu C., Zhou Z., Qiao P. (2018). A Model for Hidden Behavior Prediction of Complex Systems Based on Belief Rule Base and Power Set. IEEE Trans. Syst. Man Cybern. Syst..

[47] Li G., Zhou Z., Hu C., Chang L., Zhou Z., Zhao F. (2017). A new safety assessment model for complex system based on the conditional generalized minimum variance and the belief rule base. Saf. Sci..

[48] Feng Z., Zhou Z.J., Hu C., Chang L., Hu G., Zhao F. (2019). A New Belief Rule Base Model With Attribute Reliability. IEEE Trans. Fuzzy Syst..

